# Hypoallergenic Wheat Line (1BS-18H) Lacking ω5-Gliadin Induces Oral Tolerance to Wheat Gluten Proteins in a Rat Model of Wheat Allergy

**DOI:** 10.3390/foods11152181

**Published:** 2022-07-22

**Authors:** Yukinori Yamada, Tomoharu Yokooji, Kyohei Kunimoto, Koki Inoguchi, Ryohei Ogino, Takanori Taogoshi, Eishin Morita, Hiroaki Matsuo

**Affiliations:** 1 Department of Pharmaceutical Services, Graduate School of Biomedical and Health Sciences, Hiroshima University, Hiroshima 734-8551, Japan; ykyamada@hiroshima-u.ac.jp (Y.Y.); x5jtdzdk5@gmail.com (K.K.); b170397@hiroshima-u.ac.jp (K.I.); taogo@hiroshima-u.ac.jp (T.T.); hmatsuo@hiroshima-u.ac.jp (H.M.); 2 Department of Frontier Science for Pharmacotherapy, Graduate School of Biomedical and Health Sciences, Hiroshima University, Hiroshima 734-8553, Japan; ryogino@hiroshima-u.ac.jp; 3 Department of Dermatology, Faculty of Medicine, Shimane University, Izumo 693-8501, Japan; emorita@med.shimane-u.ac.jp

**Keywords:** gluten, ω5-gliadin, hypoallergenic wheat, oral tolerance, wheat allergy

## Abstract

The early ingestion of food can prevent the onset of food allergy related to inducing oral tolerance (OT). We developed the Hokushin wheat line as a hypoallergenic wheat (1BS-18H) lacking ω5-gliadin, a major allergen of wheat-dependent exercise-induced anaphylaxis (WDEIA). The 1BS-18H wheat had lower ability of sensitization for ω5-gliadin compared with Hokushin wheat. Here, we evaluated the induction of OT to gluten and ω5-gliadin by the early consecutive ingestion of 1BS-18H gluten using a rat model of wheat allergy. Rats were subcutaneously immunized with commercial gluten or native ω5-gliadin following the daily oral administration of gluten. The daily oral administration of 1BS-18H gluten for 5 days before immunization suppressed the increase in gluten- or ω5-gliadin-specific IgE and IgG_1_ antibodies induced by immunization to a level similar to Hokushin gluten. Intravenous challenge with gluten or ω5-gliadin did not decrease the rectal temperature in rats with OT induced by 1BS-18H or Hokushin gluten, although it was decreased in non-OT rats. In conclusion, the early consecutive ingestion of 1BS-18H wheat before sensitization induced OT to gluten and ω5-gliadin. These findings support the benefit of 1BS-18H wheat to prevent wheat allergy including WDEIA by consecutive ingestion in humans.

## 1. Introduction

Wheat is consumed as a staple food and a functional ingredient in processed foods worldwide. However, wheat can cause IgE-mediated allergies, including immediate-type wheat allergy and wheat-dependent exercise-induced anaphylaxis (WDEIA) [1,2]. Patients with IgE-mediated wheat allergy, including WDEIA, can develop life-threatening anaphylaxis. WDEIA is a special form of IgE-mediated wheat allergy. Although patients with WDEIA do not demonstrate any allergic symptoms such as dyspnea, skin manifestations or anaphylactic shock by wheat ingestion alone, they experience symptoms generated by a combination of wheat ingestion and cofactors such as physical exercise, intake of non-steroidal anti-inflammatory drugs, and intake of alcohol [3]. In Japan, the frequency of food-dependent exercise-induced anaphylaxis (FDEIA) was reported as 0.017% [4], and the frequency of WDEIA was 56% in patients with FDEIA [5]. We previously reported that ω5-gliadin was the predominant allergen in patients with various wheat allergies, especially WDEIA [6,7]. A curative treatment for wheat allergy including WDEIA has not been established. Thus, patients with wheat allergy should completely avoid exposure to wheat products to prevent the elicitation of allergic symptoms. However, the strict avoidance of wheat may decrease the quality of life (QOL) for wheat-allergic patients. Furthermore, patients have a risk of anaphylaxis by the accidental ingestion of wheat allergens because they are contained in various processed foods [8].

Recently, several studies have developed hypoallergic foods to improve the QOL of food-allergic patients. Herman et al. produced a hypoallergenic soybean lacking the major soybean allergen Gly m Bd 30 K using transgene-induced gene-silencing technology [9]. Lee et al. developed hypoallergic cow’s milk with reduced levels of bovine α-casein and β-lactoglobulin by irradiation with gamma rays [10]. Regarding wheat cultivars, Altenbach et al. produced transgenic wheat with a reduced content of ω5-gliadin using RNA interference technology [11,12,13,14]. However, these hypoallergic foods may be unacceptable to consumers, especially in Japan, because they were developed using transgenic technology [15]. Furthermore, the breadmaking properties of transgenic wheat cultivars are often reduced because of the reduced content of allergenic proteins such as gliadin and glutenin, which are responsible for the viscoelastic properties of wheat flour. We previously developed a hypoallergenic wheat line (1BS-18H) by the repeated backcrossing of the Chinese Spring wheat line 1BS-18 lacking the ω5-gliadin locus on chromosome 1B with Hokushin wheat, which is widely eaten in Japan [16]. Our new 1BS-18H wheat is not transgenic because there is no insertion of foreign genes. Our preliminary study showed that 1BS-18H wheat retained the unique viscoelastic properties of wheat for use in products such as bread and noodles by tensile test for gluten dough, and its moisture, protein, and ash content were similar to those of common wheat as reported by Ma et al. [17]. We previously reported that ω5-gliadin was detected in 1BS-18H gluten only approximately 23% of that in Hokushin gluten [18], and the intravenous challenge of 1BS-18H gluten did not elicit an allergic reaction in rats sensitized to ω5-gliadin [19].

Lack proposed the dual-allergen exposure hypothesis, which postulates that allergen exposure through the skin causes food allergy whereas the early ingestion of allergen induces immunotolerance [20]. On the basis of this hypothesis, many researchers have attempted to prevent food allergy through the acquisition of oral immunotolerance by the early active ingestion of a food allergen. Du Toit et al. reported that the early consumption of peanuts induced oral tolerance (OT) to peanuts that prevented peanut allergy in the Learning Early About Peanut Allergy (LEAP) study [21]. Perkin et al. reported that the prevalence of peanut and egg allergy was significantly lower in an early introduction group than in the standard introduction group although there were no significant effects with respect to milk, sesame, fish, or wheat in the Enquiring About Tolerance (EAT) study [22]. These reports suggest that the early ingestion of food might prevent food allergy. However, the early consecutive ingestion of food is accompanied by a risk of oral sensitization to food allergens. Our previous report showed that 1BS-18H wheat had lower sensitization for ω5-gliadin compared with Hokushin wheat [19]. However, it is unclear whether 1BS-18H wheat induces OT to ω5-gliadin because 1BS-18H wheat does not contain ω5-gliadin. We speculated that gluten prepared from 1BS-18H wheat would induce OT to ω5-gliadin by the cross-reactivity between ω5-gliadin and other gluten components because gluten components possess similar amino acid (aa) sequences [23]. In this study, we examined whether 1BS-18H gluten induced OT to gluten and ω5-gliadin to prevent wheat allergy using a rat model.

## 2. Materials and Methods

### 2.1. Materials

Commercial gluten (TCI gluten), gliadin, and glutenin were purchased from Tokyo Chemical Industry (Tokyo, Japan). Glutens were prepared from Hokushin (Koshoku; Tokyo, Japan), and 1BS-18H wheat flours were produced by Kohno et al. [16] as reported previously [18]. Native ω5-gliadin was purified from Hokushin wheat flour as reported previously [24]. Alum adjuvant (Imject^®^ Alum) and an ELISA plate (F8 MaxiSorp loose Nunc-Immuno™ Modules) were purchased from Thermo Fisher Scientific (Waltham, MA, USA). Biotin Labeling Kit-NH_2_, Block Ace^®^, and 3,3′,5,5′-tetramethylbenzidine (TMB) solution were obtained from Dojindo (Tokyo, Japan), DS Pharma Biomedical (Osaka, Japan), and SeraCare Life Sciences (Gaithersburg, MD, USA), respectively. Western blotting detection reagents (Western Lightning^®^ Ultra) were from PerkinElmer (Waltham, MA, USA). Anti-gliadin antibodies (Abs) and horseradish peroxidase (HRP)-conjugated anti-rabbit IgG were purchased from Accurate Chemical & Scientific Corporation (Carle Place, NY, USA) and Biosource (Camarillo, CA, USA), respectively. Mouse anti-rat IgE Abs (MARE-1) was purchased from GeneTex (Irvine, CA, USA). HRP-conjugated goat anti-rat IgG_1_ Abs was purchased from Bethyl Laboratories (Montgomery, TX, USA). HRP-conjugated streptavidin was obtained from Proteintech Japan (Tokyo, Japan). IgG against the ω5-gliadin epitope peptide produced in a previous study was purified using a peptide affinity column [16]. All chemicals used were of the highest purity available.

### 2.2. Western Blot Analysis against Wheat Gluten

Gluten [10 μg for Western blot and 25 μg for Coomassie brilliant blue (CBB) staining] prepared from Hokushin (Hokushin gluten) and 1BS-18H wheat flours (1BS-18H gluten) were applied to each lane of a 5.1% polyacrylamide stacking gel, and then separated in 12.5% polyacrylamide running gel. Western blot analysis was performed using anti-gliadin Abs (1:10,000) and anti-ω5-gliadin Abs (1:10,000) as primary Abs and HRP-conjugated anti-rabbit IgG as a secondary Ab following SDS-PAGE. To detect proteins bound to each Ab, Western Lightning^®^Ultra and image analyzer LAS-4000 mini (GE Healthcare, Little Chalfont, UK) were used as reported previously [16,18].

### 2.3. Animals

Three-week-old female Brown-Norway (BN) rats were purchased from Japan SLC (Shizuoka, Japan). Rats were fed water and a gluten-free diet (AIN-93G; Oriental Yeast Company, Tokyo, Japan) freely for more than 1 week before the experiments. All animal experiments were approved by the animal ethics committee of Hiroshima University (approval No. A-16-44-3).

### 2.4. Induction of OT to Gluten and Its Components in Rats

**Figure 1:**
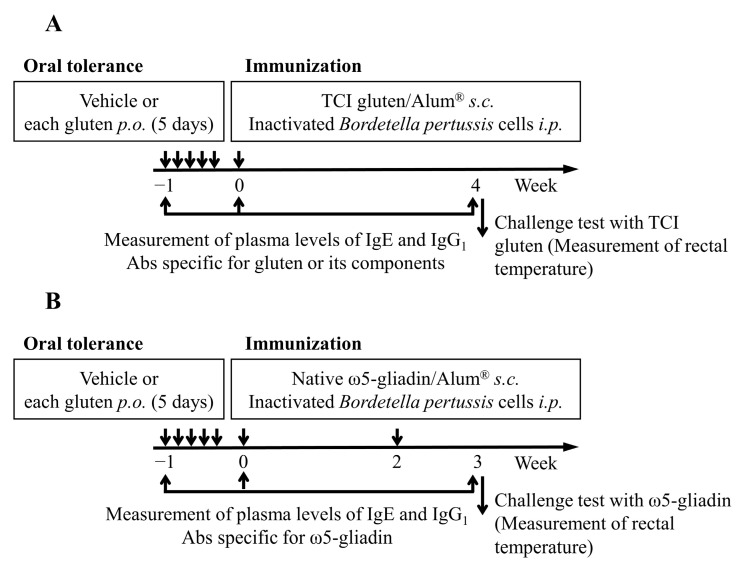
The immunization and induction of oral tolerance to gluten and its components (schematic). BN rats were orally administered with vehicle alone (50% ethanol) or 10 mg of each type of gluten daily for 5 days for OT induction. At 2 days after the final oral administration (week 0), rats were administered subcutaneously once with TCI gluten for immunization with gluten (**A**). For immunization with ω5-gliadin, rats were administered subcutaneously with ω5-gliadin at weeks 0 and 2 (**B**). Blood was collected to measure the plasma levels of IgE and IgG_1_ Abs specific for gluten or its components at weeks −1, 0, and 3 (for ω5-gliadin study) or 4 (for gluten study). Rectal temperatures were measured after challenge with TCI gluten or ω5-gliadin at week 3 or 4 intravenously. *p.o.*, *per os* (oral administration); *s.c.*, subcutaneous administration; *i.p.*, intraperitoneal administration.

To evaluate the effect of each gluten on the induction of OT to gluten and its components, an OT study was performed in accordance with the method reported by Kumagai et al. [25] with a slight modification as shown in Figure 1. Briefly, 10 mg of TCI gluten, Hokushin gluten, or 1BS-18H gluten was suspended in physiological saline containing 50% ethanol (0.2 mL). Each gluten suspension (0.2 mL, OT) or vehicle alone (0.2 mL, non-OT) was administered orally to rats daily using a stainless-steel feeding tube for 5 days. At 2 days after the final oral administration (week 0), rats were immunized by subcutaneous injection with physiologic saline (0.9% NaCl, 0.5 mL) containing 5 mM acetic acid, 2 mg/mL of TCI gluten, and Imject^®^ Alum [10 mg/mL of Al(OH)_3_ and 10 mg/mL of Mg(OH)_2_] once (Figure 1A). For ω5-gliadin, rats were immunized subcutaneously with physiologic saline (0.5 mL) containing 5 mM acetic acid, 10 mg/mL of native ω5-gliadin, and Imject^®^ Alum [10 mg/mL of Al(OH)_3_ and 10 mg/mL of Mg(OH)_2_] at weeks 0 and 2 (Figure 1B). These rats were intraperitoneally administered PBS containing inactivated *Bordetella pertussis* cells (6 × 10^8^ cells, 0.2 mL) at immunization. At weeks −1, 0, and 3 for ω5-gliadin or week 4 for gluten studies, blood (300 µL) was collected from the jugular vein to determine the levels of gluten or its component-specific IgE and IgG_1_ Abs in plasma using ELISA.

### 2.5. Measurement of Plasma Levels of IgE and IgG_1_ Abs Specific for Gluten and Its Components

To evaluate the sensitization to gluten and its components, plasma levels of IgE and IgG_1_ Abs specific for gluten, gliadin, glutenin, and ω5-gliadin were determined by an ELISA in accordance with our previous report [26] with a modification. To determine the levels of gluten and its component-specific IgE Abs in plasma, each well of an ELISA plate was coated with 1 µg/mL MARE-1 dissolved in PBS (100 µL, pH 7.4) overnight at 4 °C. After washing with PBS containing 0.1% Tween 20 (PBS-T) six times, plates were incubated with 1% Block Ace^®^ for 1 h at 37 °C. Then, each rat plasma sample (100 μL, diluted 1:10 in 10% Block Ace^®^) was added to each well. After incubation for 2 h at 37 °C, each well was washed with PBS-T. Then, the wells were incubated with TCI gluten, gliadin, glutenin, or native ω5-gliadin labeled with biotin using a Biotin Labeling Kit-NH_2_ (100 µL, diluted 1:1000 in 1% Block Ace^®^) for 2 h at 37 °C. Wells were washed with PBS-T and then incubated with HRP-conjugated streptavidin (100 µL) for 1 h at 37 °C. After washing with PBS-T, wells were incubated with TMB (100 µL) at 37 °C. After a 15-min incubation, the reaction was terminated with 1 M phosphoric acid (100 µL). Absorbance was measured at 450 nm against 630 nm as a reference using a Multiskan GO spectrophotometer (Thermo Fisher Scientific).

To determine the levels of gluten and its component-specific IgG_1_ in plasma, each well of an ELISA plate was coated with TCI gluten (100 µL, 10 µg/mL) or native ω5-gliadin (100 µL, 20 µg/mL) dissolved in 0.02 M acetic acid overnight at 4 °C. After blocking, each rat plasma sample (100 µL, diluted 1:10,000–30,000 in 1% Block Ace^®^) was added to each well. After incubation for 1 h at room temperature, the wells were washed and incubated with HRP-conjugated goat anti-rat IgG_1_ Abs (100 µL, diluted 1:100,000 in PBS) for 1 h at room temperature. The wells were washed with PBS-T and then incubated with TMB (100 µL) for 15 min at room temperature. The reaction was terminated with 1 M phosphoric acid (100 µL), and the absorbance was measured at 450 nm against 630 nm as a reference.

### 2.6. Evaluation of Systemic Anaphylaxis

Systemic anaphylaxis was assessed by measuring changes in rectal temperature for 30 min after an administration of TCI gluten or ω5-gliadin intravenously at week 3 for ω5-gliadin or week 4 for gluten studies following a previous report [27]. In this study, TCI gluten or ω5-gliadin was intravenously administered because their allergenicities could be evaluated while excluding the processes of digestion and absorption. The rectal temperature was monitored using a specific rectal thermometer for rats (Shibaura Electronics, Saitama, Japan) before and 30 min after the administration of each wheat protein intravenously. The rectal temperature was measured at 0 min, and then TCI gluten (2 mg/kg) or native ω5-gliadin (2 mg/kg) was administered intravenously via the jugular vein. In this study, the rectal temperature was measured every 5 min for 30 min.

### 2.7. Statistical Analyses

Data are shown as the mean ± standard deviation (SD) of the mean. Differences in the mean values between groups were assessed using Mann–Whitney U test or Kruskal–Wallis test, followed by a nonparametric post hoc Steel–Dwass test. We considered *p* < 0.05 statistically significant. Statical analysis was performed using the Statcel4 software (version 1.0, OMS Publishing Inc., Saitama, Japan).

## 3. Results

### 3.1. Western Blot Analysis against Wheat Gluten

**Figure 2:**
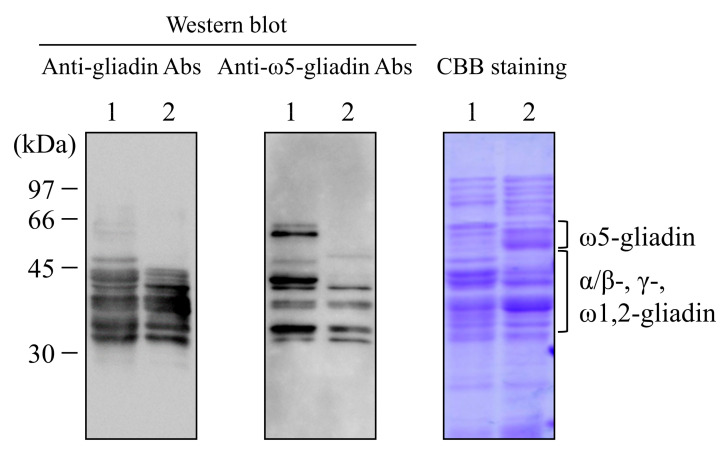
Western blot analysis of the gluten from two wheat cultivars. Gluten (10 μg) from Hokushin (lane 1) and 1BS-18H wheat (lane 2) was separated by SDS-PAGE and blotted onto a PVDF membrane. The membrane was incubated with anti-gliadin Abs and anti-ω5-gliadin Abs. For CBB staining, 25 μg of each gluten was loaded onto the gel.

The gluten components in the Hokushin and 1BS-18H glutens were confirmed by Western blot analysis with anti-gliadin and anti-ω5-gliadin Abs (Figure 2). Bands corresponding to the molecular sizes of α/β-, γ-, and ω1,2-gliadins (35–50 kDa) were observed in the Hokushin and 1BS-18H glutens using anti-gliadin Abs, but faint bands of ω5-gliadin (~60 kDa) were observed only in the Hokushin gluten. When we used anti-ω5-gliadin Abs to detect the gluten components, we obtained strong bands of ω5-gliadin only in the Hokushin gluten, although bands of α/β-, γ-, and ω1,2-gliadins were observed in the Hokushin and 1BS-18H glutens. Thus, ω5-gliadin protein was not detected in the gluten components of 1BS-18H.

### 3.2. Induction of OT to Gluten in Rats

**Figure 3:**
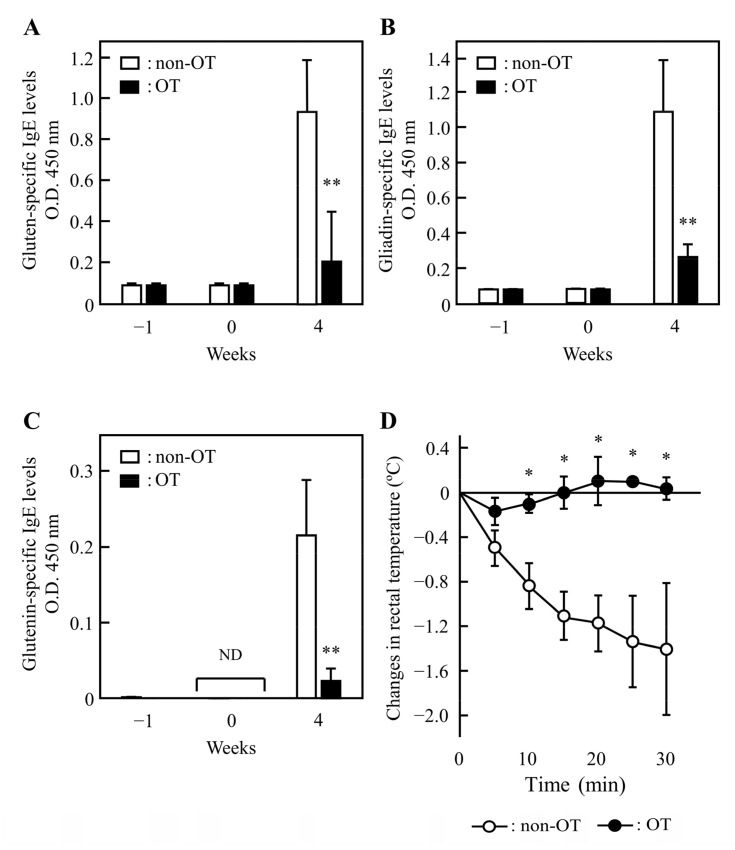
The induction of oral tolerance (OT) to gluten and its components in gluten-immunized rats. The levels of IgE Abs specific for gluten (**A**), gliadin (**B**), and glutenin (**C**) in plasma were determined at weeks −1, 0, and 4 in rats immunized subcutaneously with gluten (week 0) following the oral administration of vehicle alone (50% ethanol, non-OT) or 10 mg of TCI gluten (OT) daily for 5 days. Changes in rectal temperature (**D**) were evaluated after the administration of gluten (2 mg/kg) intravenously at week 4. Each value represents the mean ± SD of three to six rats. * *p* < 0.05, ** *p* < 0.01: significantly different from non-OT rats. ND, not detected.

To confirm whether our rat model of wheat allergy was suitable for evaluating the induction of OT to gluten, we measured the plasma levels of gluten-specific IgE and IgG_1_ Abs in a rat model of wheat allergy. In non-OT rats, plasma levels of gluten-specific IgE and IgG_1_ Abs were increased at 4 weeks after subcutaneous immunization with gluten. The plasma IgE levels specific for gluten that increased after immunization with gluten were significantly suppressed when rats were orally administered gluten daily for 5 days before the immunization (Figure 3A). In addition, the oral administration of gluten before the immunization slightly suppressed the increased plasma levels of IgG_1_ Abs specific for gluten (Figure S1A). To further evaluate the induction of OT to gluten components in these rats, we measured the plasma levels of gliadin- and glutenin-specific IgE and IgG_1_ Abs. Similar to the IgE and IgG_1_ Abs specific for gluten, the IgE and IgG_1_ Abs levels specific for gliadin and glutenin were increased by immunization with gluten in non-OT rats at 4 weeks. The increased levels of IgE and IgG_1_ Abs specific for gliadin (Figure 3B and Figure S1B) and glutenin (Figure 3C and Figure S1C) were suppressed by the consecutive oral administration of gluten before the immunization.

To confirm the effect of OT induction on anaphylactic reactions in our rat model, we monitored the rectal temperature for 30 min following the intravenous injection with gluten at 4 weeks after immunization. When gluten was administered intravenously in non-OT rats, their temperature decreased by 1.4 °C at 30 min (Figure 3D). Intravenous injection with gluten exerted no change in the temperature of OT rats. Thus, our rat model of wheat allergy is suitable to evaluate the induction of OT to gluten.

### 3.3. Effect of 1BS-18H Gluten on the Induction of OT to Normal Gluten

**Figure 4:**
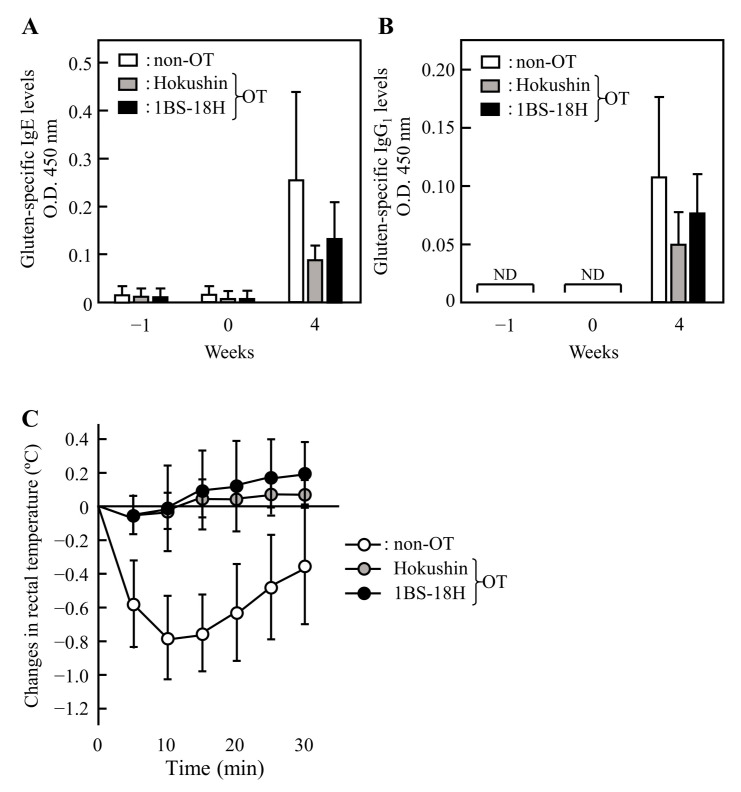
The effects of the oral administration of 1BS-18H gluten on the induction of oral tolerance (OT) to gluten in gluten-immunized rats. The levels of IgE (**A**) and IgG_1_ (**B**) Abs specific for gluten in plasma were determined at weeks −1, 0, and 4 in rats immunized subcutaneously with gluten (week 0) following the oral administration of vehicle alone (50% ethanol, non-OT), gluten from Hokushin (10 mg, Hokushin), or gluten from 1BS-18H (10 mg, 1BS-18H) daily for 5 days. Changes in rectal temperature (**C**) were evaluated after the administration of gluten (2 mg/kg) intravenously at week 4. Each value represents the mean ± SD of four to eight rats. ND, not detected.

To examine the effect of 1BS-18H gluten on the induction of OT to normal gluten, we measured the plasma levels of IgE and IgG_1_ Abs specific for TCI gluten in rats immunized with TCI gluten following the oral administration of 1BS-18H gluten daily for 5 days. In this study, Hokushin gluten was administered to rats instead of 1BS-18H gluten as a positive control. The oral administration of Hokushin gluten before the immunization tended to suppress the plasma levels of gluten-specific IgE and IgG_1_ Abs that were increased by immunization (Figure 4A,B). The oral administration of 1BS-18H gluten suppressed the elevation of gluten-specific IgE and IgG_1_ Abs to the same degree as Hokushin gluten (Figure 4A,B), although there was no significant difference statistically.

Next, we measured the rectal temperature in rats with non-OT or OT induced by Hokushin and 1BS-18H gluten after intravenous challenge with TCI gluten (Figure 4C). In the non-OT rats, intravenous injection with gluten reduced the temperature by 0.8 °C at 10 min. Intravenous injection with gluten exerted no reduction in the rectal temperature of rats with OT induced by 1BS-18H gluten or Hokushin gluten. Thus, the oral administration of 1BS-18H gluten induced OT to normal gluten before immunization.

### 3.4. Effect of 1BS-18H Gluten on the Induction of OT to ω5-Gliadin

**Figure 5:**
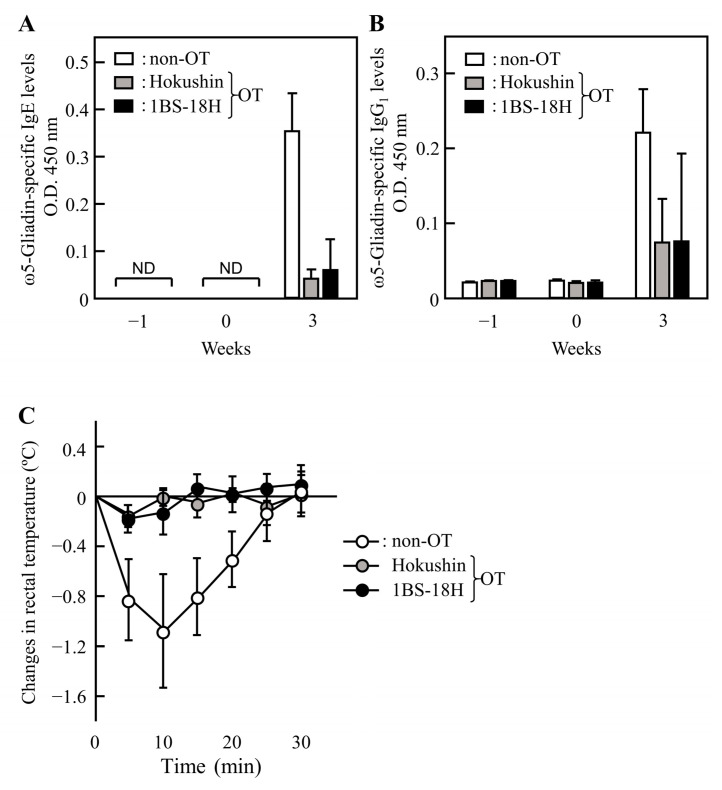
The effects of the oral administration of 1BS-18H gluten on the induction of oral tolerance (OT) to gluten in ω5-gliadin-immunized rats. The levels of IgE (**A**) and IgG_1_ (**B**) Abs specific for ω5-gliadin in plasma were determined at weeks −1, 0, and 3 in rats immunized subcutaneously with ω5-gliadin (weeks 0 and 2) following the oral administration of vehicle alone (50% ethanol, non-OT), gluten from Hokushin (10 mg, Hokushin), or gluten from 1BS-18H (10 mg, 1BS-18H) daily for 5 days. Changes in rectal temperature (**C**) were evaluated after the administration of ω5-gliadin (2 mg/kg) intravenously at week 3. Each value represents the mean ± SD of four rats. ND, not detected.

To examine whether 1BS-18H gluten induced OT to ω5-gliadin, we measured the levels of ω5-gliadin-specific IgE and IgG_1_ Abs in rat plasma immunized with ω5-gliadin following the daily oral administration of 1BS-18H gluten and Hokushin gluten for 5 days. As shown in Figure 5A,B, the elevation of ω5-gliadin-specific IgE and IgG_1_ Abs was slightly induced by the oral administration of Hokushin gluten before the immunization. In addition, the oral administration of 1BS-18H gluten induced low ω5-gliadin-specific IgE and IgG_1_ Abs levels. Although intravenous injection with ω5-gliadin reduced the rectal temperature by 1.1 °C at 10 min in non-OT rats, injection with ω5-gliadin did not alter the rectal temperature in rats with OT induced by gluten from 1BS-18H wheat, similar to Hokushin wheat (Figure 5C); however, there was no significant difference statistically. These results suggest that 1BS-18H gluten induces OT to ω5-gliadin even though 1BS-18H gluten does not contain ω5-gliadin.

## 4. Discussion

Many previous reports have shown that the early ingestion of food allergens for infants during the early-weaning period prevented the development of food allergy since the dual–allergen exposure hypothesis was proposed by Lack [20,21,22]. However, the possibility that the early consecutive ingestion of food allergens can cause the development of food allergy through oral sensitization has not been ruled out. In this study, we demonstrated that 1BS-18H wheat lacking ω5-gliadin induced OT to normal gluten and ω5-gliadin using a rat model of wheat allergy. In addition, we previously showed that 1BS-18H gluten had lower ability of sensitization for ω5-gliadin than Hokushin gluten [19]. Our wheat, 1BS-18H, is not transgenic, and it retains unique viscoelastic properties for use in wheat products. Thus, the ingestion of 1BS-18H wheat in early life in healthy individuals (before sensitization to ω5-gliadin) might prevent the induction of wheat allergy, especially ω5-gliadin-sensition type allergies such as WDEIA.

Western blot analysis showed that ~60-kDa bands for ω5-gliadin were not detected in 1BS-18H gluten using IgG Abs for ω5-gliadin, although the bands were present in the Hokushin gluten sample, indicating that 1BS-18H gluten does not contain ω5-gliadin protein. Our previous reports showed that 1.21 mg/g and 5.17 mg/g of ω5-gliadin were detected in 1BS-18H gluten and Hokushin gluten by ELISA, respectively [18]. In that study, however, we used anti-ω5-gliadin Abs produced using the peptide KQQSPEQQQFPQQQIPQQQ, including three IgE-binding epitope sequences of ω5-gliadin, QQIPQQQ, QQFPQQQ, and QQSPEQQ to detect ω5-gliadin [16]. Cassidy et al. reported that several gliadins and low-molecular-weight glutenin contain common epitope sequences such as the QQFPQQQ sequence of ω5-gliadin [28]. Indeed, the same anti-ω5-gliadin Abs strongly bound to several gluten components in addition to ω5-gliadin (Figure 2). Thus, we consider that its slight detection in 1BS-18H gluten may be related to the cross-reactivity of anti-ω5-gliadin Abs to other gluten components in ELISA. Altenbach et al. reported that a mutant wheat line lacking the major ω5-gliadin encoded on chromosome 1B had several minor ω5-gliadin proteins located on chromosome 1D that possessed several IgE-binding epitopes for WDEIA patients [12]. This report suggests that 1BS-18H wheat may also contain minor ω5-gliadin encoded on chromosome 1D. However, intravenous injection with 1BS-18H gluten exerted no decrease in the rectal temperature in rats immunized with ω5-gliadin [19]. This suggests that 1BS-18H gluten does not contain ω5-gliadin encoded on chromosome 1D at a level sufficient to elicit anaphylaxis in vivo.

The levels of gluten-specific IgE and IgG_1_ Abs in plasma increased after gluten-immunization were suppressed when rats were orally administered gluten daily for 5 days before the immunization (Figure 3A and Figure S1A). In these rats, the levels of gliadin- and glutenin-specific IgE and IgG_1_ Abs in plasma that increased by immunization with gluten were also suppressed by the oral administration of gluten before the immunization (Figure 3B,C and Figure S1B,C). We confirmed that the oral administration of gliadin and glutenin before immunization with each component suppressed the plasma levels of IgE and IgG_1_ Abs specific for each component that were increased by immunization (data not shown). Thus, the reduction of gluten-specific IgE and IgG_1_ Ab levels in plasma by the oral administration of gluten was ascribed to the induction of OT to gliadin and glutenin contained in gluten. Intravenous injection with TCI gluten reduced the rectal temperature of non-OT rats but not in rats with OT to gluten (Figure 3D), indicating that our rat model of wheat allergy was suitable for evaluating the induction of oral immunotherapy (OIT) to gluten. The mechanisms for OT induction are poorly understood. It is thought that ingested food allergens are recognized by a subset of regulatory dendritic cells expressing CD103 that induce regulatory lymph cells such as Treg specific to the allergen, resulting in OT [29,30]. However, the mechanism of OT induction might be affected by the dose of allergen and the frequency of administration. Chen et al. reported that five oral administrations of high-dose (500 mg) OVA induced anergy or T-cell depletion, whereas low-dose (0.5 mg) OVA induced Treg in a mouse model [31]. Our preliminary flow cytometric analysis showed that the relative cell populations of FoxP3+CD4+CD25+ Treg in mesenteric lymph nodes and spleens from rats with OIT to gluten were 1.1–1.4-fold higher than those from non-OIT rats at 4 weeks after immunization (data not shown). Thus, we speculated that the induction of Treg may be in part associated with the development of OT to gluten in our rat model. Further studies are necessary to clarify the mechanisms of the induction of OT to gluten in our rat model.

The consecutive oral administration of 1BS-18H gluten before the immunization with gluten suppressed the elevation of gluten-specific IgE and IgG_1_ Abs to the same degree as Hokushin gluten (Figure 4A,B). In addition, the rectal temperature in rats with OT to gluten induced by oral 1BS-18H gluten, similar to Hokushin gluten, was not reduced by intravenous injection with gluten. These results suggest that 1BS-18H and Hokushin gluten can induce OT to gluten before immunization. Furthermore, the consecutive oral administration of 1BS-18H gluten also induced OT to ω5-gliadin even though this wheat cultivar did not contain ω5-gliadin (Figure 5). Why 1BS-18H gluten induced OT to ω5-gliadin is unclear. We speculate that 1BS-18H gluten induced OT to ω5-gliadin as a result of cross-reactivity between ω5-gliadin and other gluten components related to their similar aa sequences, which induce Treg against ω5-gliadin. This potential cross-reactivity is supported by experiments in which IgE Abs from patients with wheat allergy strongly bound to the QQX_1_PX_2_QQ consensus motif found in ω5-gliadin and other gliadins (where X_1_ is L, F, S, or I and X_2_ is Q, E, or G) [32,33]. In this study, we did not identify the aa sequences of T-cell epitopes in ω5-gliadin that induced OT or Treg against ω5-gliadin, and these should be determined in future studies using our rat model of wheat allergy.

OIT is performed as a curative treatment for wheat allergy [34]. However, it is difficult to perform OIT using wheat allergens because IgE-mediated allergic symptoms can be triggered by the ingestion of allergens in patients with wheat allergy. To prevent the elicitation of allergic symptoms in patients, hypoallergenic wheat allergens modified by enzymes [35,36] or resin [25,37] are available for OIT. We previously demonstrated that the intravenous administration of 1BS-18H gluten did not elicit an allergic reaction in rats immunized with ω5-gliadin [19]. Thus, 1BS-18H wheat can be used for OIT with no side effects including anaphylactic reaction for ω5-gliadin-sensitized types of wheat-allergic patients, including WDEIA patients.

In this study, we demonstrated that the early ingestion of 1BS-18H wheat before immunization induced OT to normal gluten and ω5-gliadin in a rat model of wheat allergy. However, the limitation of this study was that we could not determine how effective early ingestion of 1BS-18H wheat was in the induction of OT to gluten or ω5-gliadin from common wheat in healthy human subjects. Further studies are necessary to determine the efficacy of 1BS-18H wheat in preventing the development of ω5-gliadin-sensitized types of wheat allergy, including WDEIA in healthy human subjects.

## 5. Conclusions

We demonstrated that the early ingestion of 1BS-18H wheat before immunization induced OT to normal gluten and ω5-gliadin using a rat model of wheat allergy. 1BS-18H gluten had lower ability of sensitization for ω5-gliadin compared with that of Hokushin gluten [19] and retained its unique viscoelastic properties for use in wheat products. These findings shed new light on the prevention of wheat allergy, including ω5-gliadin-sensitized type WDEIA.

## Data Availability

Data is contained within the article or supplementary material.

## Supplementary Materials

The following supporting information can be downloaded at: https://www.mdpi.com/article/10.3390/foods11152181/s1, Figure S1: Induction of oral tolerance (OT) to gluten and its components in gluten-immunized rats.

## Abbreviations

aa: amino acid; Ab: antibody; BN, Brown-Norway; CBB, Coomassie brilliant blue; EAT, the Enquiring About Tolerance; FDEIA, food-dependent exercise-induced anaphylaxis; Hokushin gluten, gluten prepared from Hokushin wheat; HRP, horseradish peroxidase; i.p., intraperitoneal administration; i.v., intravenous administration; LEAP, Learning Early About Peanut Allergy; ND, not detected; OT, oral tolerance; PBS-T, PBS containing 0.1% Tween 20; p.o., per os; QOL, quality of life; s.c., subcutaneous administration; SD, standard deviation of the mean; TMB, 3,3′,5,5′-tetramethylbenzidine; WDEIA, wheat-dependent exercise-induced anaphylaxis; 1BS-18H, hypoallergenic Hokushin wheat line lacking ω5-gliadin; 1BS-18H gluten, gluten prepared from 1BS-18H.
