# Supplementary figures and images for: Hypoallergenic Wheat Line (1BS-18H) Lacking ω5-Gliadin Induces Oral Tolerance to Wheat Gluten Proteins in a Rat Model of Wheat Allergy
Source: Foods. 2022 Jul 22;11(15):2181. doi: 10.3390/foods11152181 (PMC9329752; doi:10.3390/foods11152181)

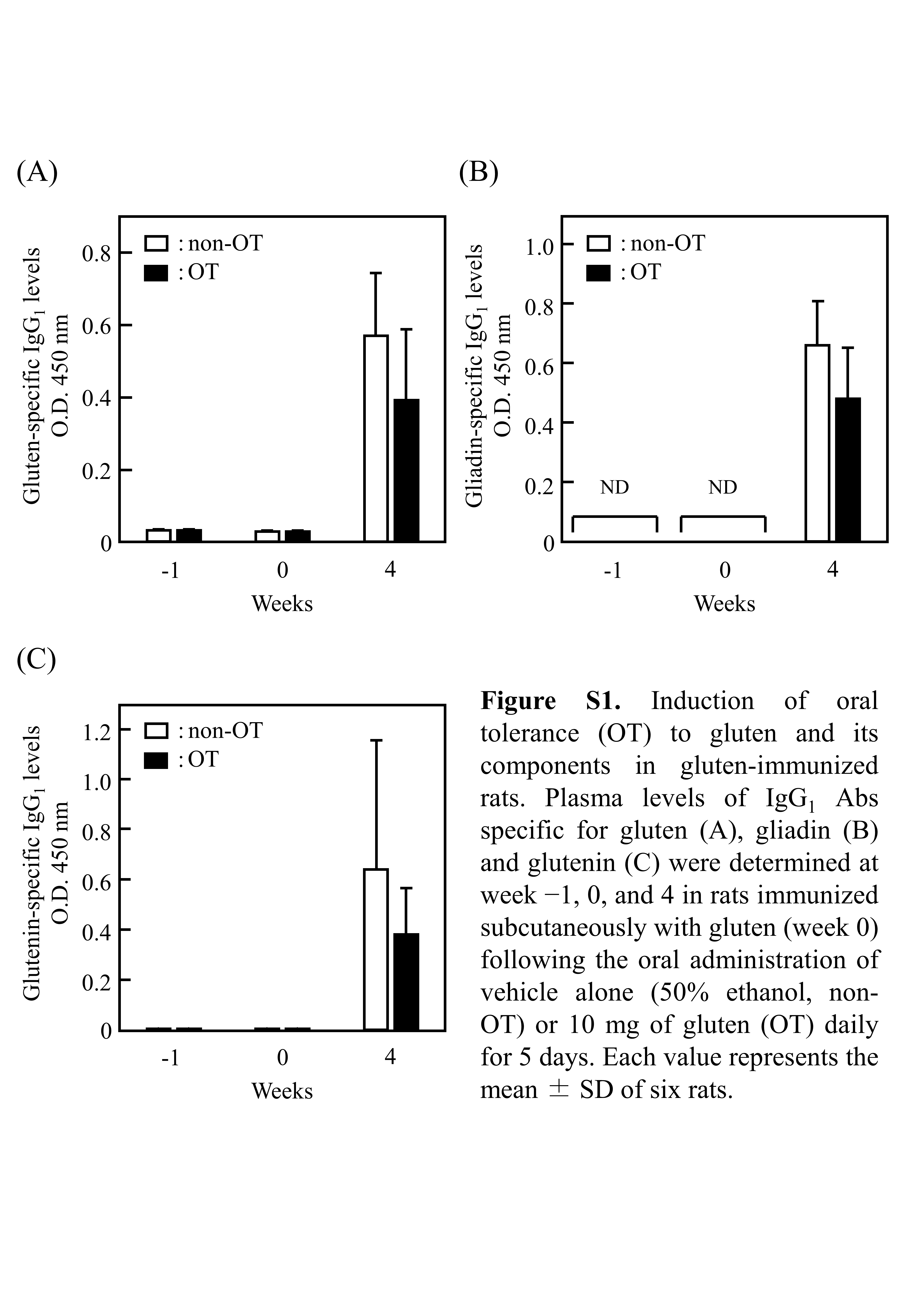

Supplement: Supplementary file 1 [file foods-11-02181-s001.zip › Supplementary Figure S1.TIF]
